# PTEN/FOXO3/AKT pathway regulates cell death and mediates morphogenetic differentiation of Colorectal Cancer Cells under Simulated Microgravity

**DOI:** 10.1038/s41598-017-06416-4

**Published:** 2017-07-20

**Authors:** Raj Pranap Arun, Divya Sivanesan, Prasanna Vidyasekar, Rama Shanker Verma

**Affiliations:** 1 Stem Cell and Molecular Biology Laboratory, Bhupat and Jyoti Mehta School of Biosciences, Department of Biotechnology, Indian Institute of Technology Madras, Chennai, 600036 India; 2 CSI-NUS, Singapore, Singapore

## Abstract

Gravity is a major physical factor determining the stress and strain around cells. Both in space experiments and ground simulation, change in gravity impacts the viability and function of various types of cells as well as *in vivo* conditions. Cancer cells have been shown to die under microgravity. This can be exploited for better understanding of the biology and identification of novel avenues for therapeutic intervention. Here, we described the effect of microgravity simulated using Rotational Cell Culture System-High Aspect Ratio Vessel (RCCS-HARV) on the viability and morphological changes of colorectal cancer cells. We observed DLD1, HCT116 and SW620 cells die through apoptosis under simulated microgravity (SM). Gene expression analysis on DLD1 cells showed upregulation of tumor suppressors PTEN and FOXO3; leading to AKT downregulation and further induction of apoptosis, through upregulation of CDK inhibitors CDKN2B, CDKN2D. SM induced cell clumps had elevated hypoxia and mitochondrial membrane potential that led to adaptive responses like morphogenetic changes, migration and deregulated autophagy, when shifted to normal culture conditions. This can be exploited to understand the three-dimensional (3D) biology of cancer in the aspect of stress response. This study highlights the regulation of cell function and viability under microgravity through PTEN/FOXO3/AKT pathway.

## Introduction

Colorectal cancer (CRC) is among the leading cause of cancer deaths worldwide and major health concern^1^. The failure of treatment of CRC is mainly due to the lack of information on its complexity in multi-factorial heterogeneity in mutations and microenvironment that cumulatively drive the survival strategy of CRC. The unique environment involving lining of functional endothelial cells in gastrointestinal tract adds value to the need of understanding the niche and physical forces involved in driving these tumors. Mechanical stimuli and stress has been shown to affect cell behavior in healthy and pathological conditions^2^. Especially in the process of metastasis and cancer stemness, physical factors of interstitial fluid pressure and matrix stiffness play a major role^3, 4^. Information about the effect of physical factors to cells on a three-dimensional (3D) scale is minimal, and that regarding the influence of gravity on the disease condition is negligible.

The role of gravity in determining cellular function and properties is more clearly depicted in the microgravity condition, which induces muscle atrophy and immune dysfunction and various other ailments in astronauts^5^. The change in gravity affects different cell types differently with either increase or decrease in function and viability^6^. Microgravity induces cell clumps and is a robust model for developing scaffold assisted and scaffold free 3D culture^7, 8^. Jessup J. M. *et al*. showed increased cell adhesion and structural formations along with elevated carcinoembryonic antigen (CEA) in mip101^9^. Ground simulation is attained using Rotational Cell Culture System- High Aspect Ratio Vessel (RCCS-HARV), Clinorotation and Random positioning^8–10^, moreover Simulated Microgravity (SM) has been shown to influence apoptosis in various cancer types, mostly through cytoskeleton dysfunction^11^. In our previous study, we observed cell death of DLD1 cells when subjected to SM, also we showed a wide array of genes deregulated, with cell cycle genes downregulated and transcriptional regulation genes upregulated^12^. We observed a possible role of AKT reduction under microgravity from the microarray.

AKT activation is related to poor prognosis in CRC^13^, also it is the driver for cell survival mechanisms contributing to cancer progression and metastasis^14^. PTEN inhibits AKT activation and subsequent nuclear translocation, activating FOXO resulting in tumor suppression^15^. This favors autophagy mediated stress adaptation, a key process in drug resistance in CRC^16^. Thus, we hypothesize that PTEN-FOXO3 axis is critical in mediating stress signaling in the scaffold free 3D spheroids from microgravity. Further, we have evaluated the role of tumor suppressors under 3D microenvironment in SM, their role in mediating stress induced cell death and adaptive measures during stress removal.

## Results

### Simulated Microgravity (SM) induces cell clumping

**Figure 1:**
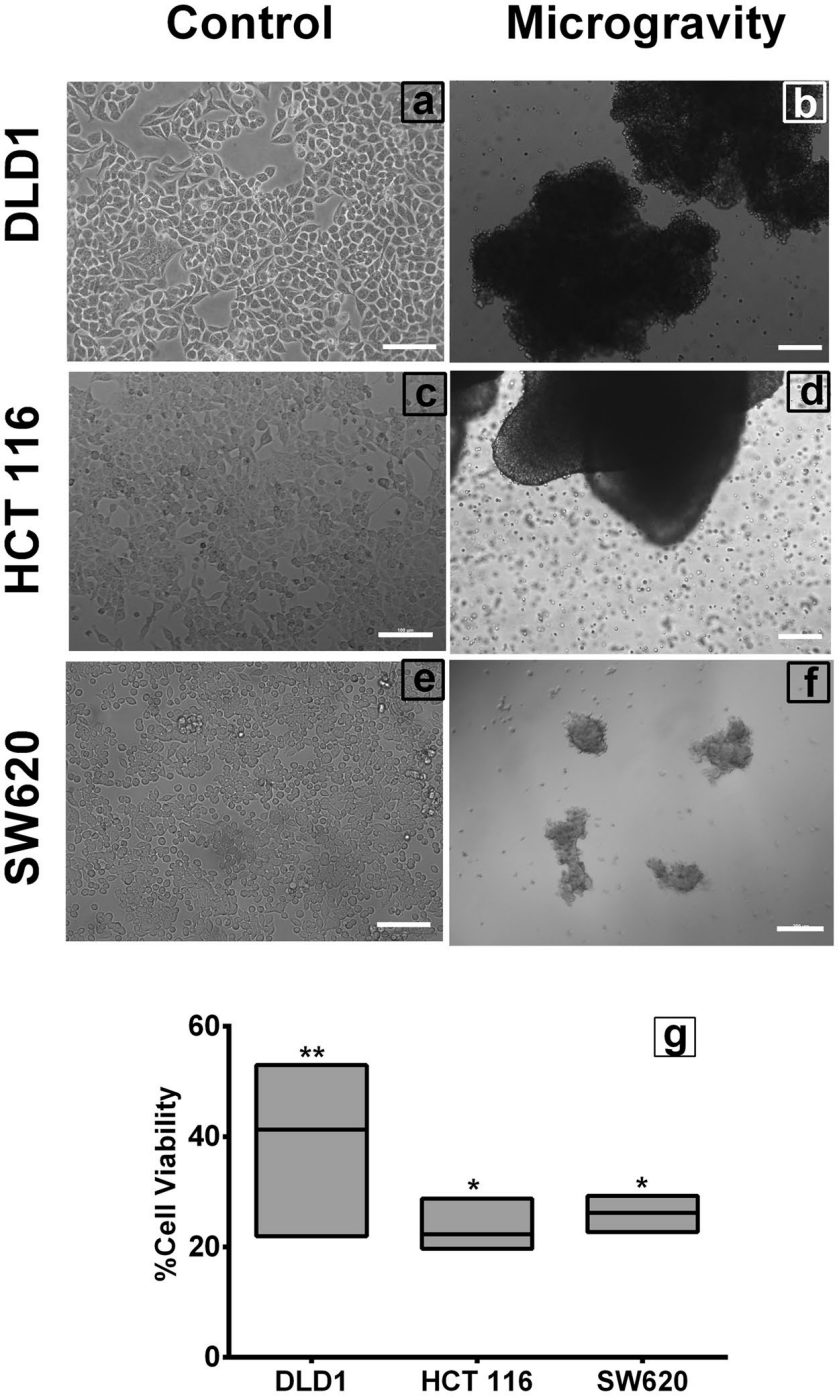
Simulation of microgravity induces cell clumping. The colorectal cancer cells DLD1 (**a**), HCT116 (**c**) and SW620 (**e**), when cultured in Rotary cell culture system at 10 RPM for 48 hours to simulate microgravity clump together to form large 3D structures (**b**,**d**,**f**) DLD1, HCT116 and SW620 respectively. Scale bar represents 200 μm. XTT assay of the microgravity simulated cells (**g**) shows the cell viability was significantly reduced. The experiment was performed thrice with individual controls **P < 0.005, *P < 0.05. Data represented as mean + S.D.

Colorectal cancer cells subjected to microgravity start to form miniscule clumps at 6–8 hours, which increase in size and form spheroids of ~200 μm to ~1 mm, which clump together to form massive 3D structures with loose arrangement of cells. By 48 hours, DLD1 and HCT116 cells formed such clumps of ~0.5 cm diameter, while SW620 cells formed smaller clumps of ~200 μm (Fig. 1a–f) (Supplementary Fig. 1). These large 3D aggregates come off spontaneously while handling and dissociate into smaller spheroids in culture. This observation parallels with the results of space and ground experiments which have shown microgravity induced clumping in various cell types^5–7, 12^. The clumps were mostly intertwined with thread like appendages along which cells roll and form the clumps and spheroids.

### Colorectal cancer cells die in microgravity

Different cell types exhibit different growth features in microgravity, mostly responding to the makeup of the cell and the physical environment of natural growth. Microgravity induces cell death in most cancer cell types^5–12^. In our experiment, we observed cell death in all the CRC cell types studied.

**Figure 2:**
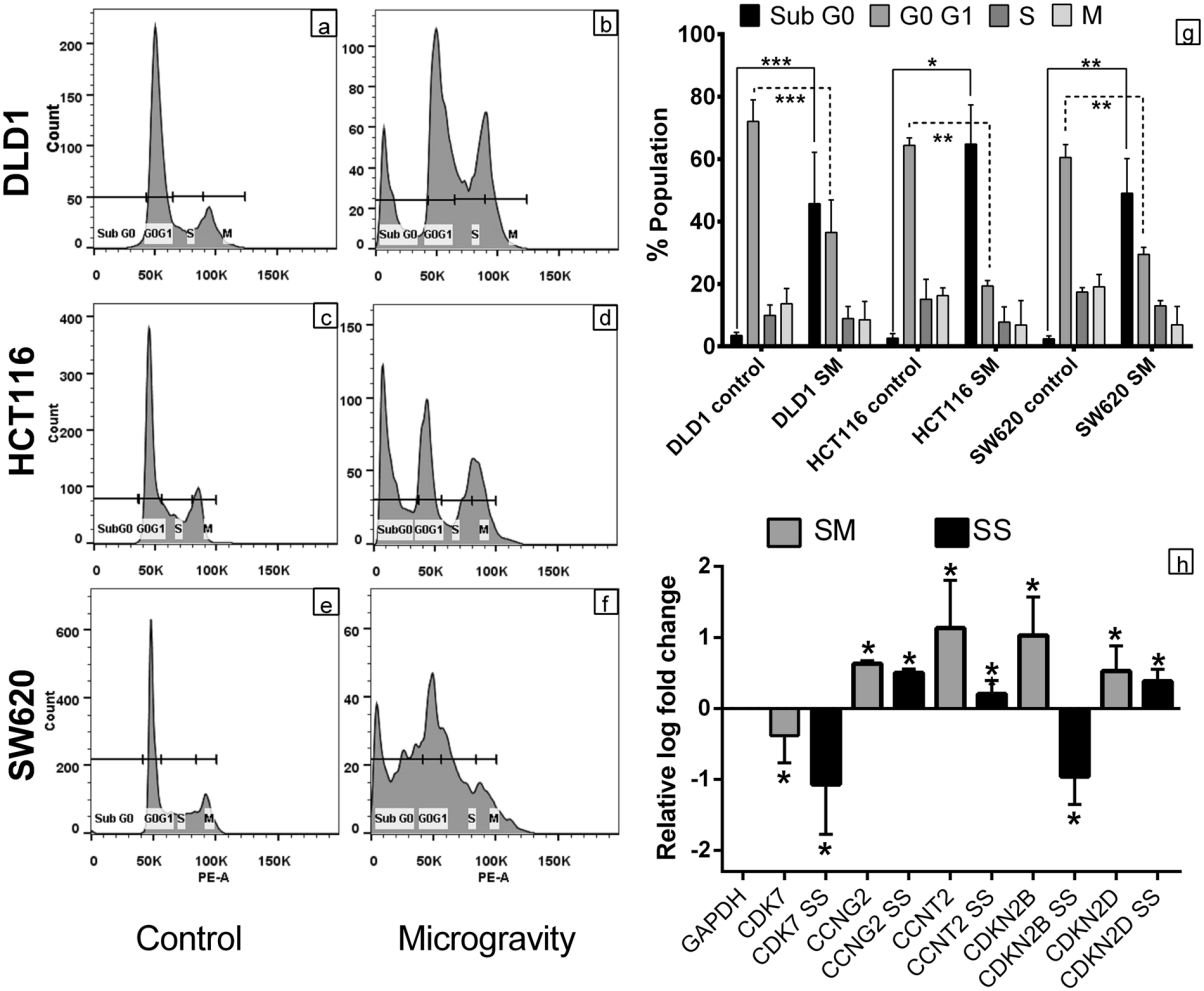
Microgravity significantly increases Sub-G0 population. Microgravity alters Cell cycle in colorectal cancer cells. The individual histogram of the Propidium iodide staining in cells counted through Flow cytometer (**a**–**f**), shows clear shift towards Sub-G0 population in SM colorectal cancer cells DLD1, HCT116 and SW620 (**b**,**d,f**) respectively, compared to the respective controls (**a**,**c**,**e**). The graphical representation of the population shift during microgravity (**g**) in mean + S.D., Sub-G0 and G0-G1 shift in DLD1 was highly significant P < 0.0005, compared to HCT116 P < 0.05 and SW620 P < 0.005. The experiment was performed a minimum of three times with individual controls. qPCR analysis of Gene expression for *CDK7, CCNG2*, *CCNT2, CDKN2B and CDKN2D* genes between DLD1 cells subjected to SM and shifted to normal (SS) with *GAPDH* as housekeeping control (**e**), represented in log fold change of mean + S.D. *P < 0.05. The experiments were performed three times with individual controls.

From viability assay, ~20% viability was observed in SW620 and HCT116 cells whereas around ~40% viability was seen in DLD1 cells simulated with microgravity (Fig. 1g). Cell cycle analysis revealed that cell death is through G1 cell cycle arrest, subsequent significant Sub G0 population (>40%) in all cell types subjected to SM. A moderate reduction in S and G2M phases were observed, and the cell death corresponds to the G1 phase arrest (Fig. 2a–f,g).

**Figure 3:**
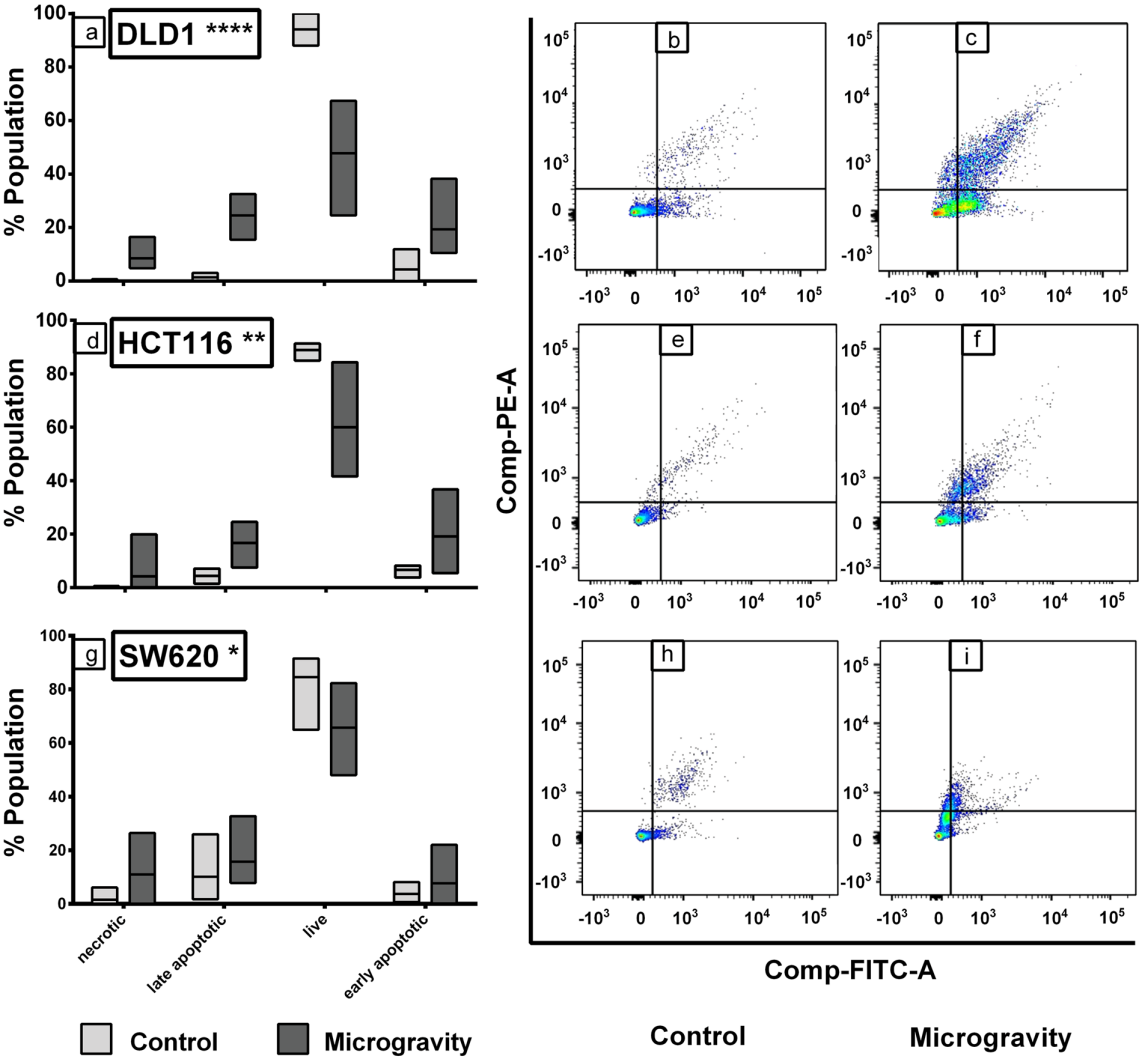
Cell death in microgravity is majorly through apoptosis. The box plot for the AnnexinV FITC & PI staining for DLD1 (**a**), HCT116 (**d**) and SW620 (**g**) shows that major cell death during SM is induced through apoptosis. The lighter boxes represent control populations and darker ones represent SM cell populations. The data is represented as mean with data range. ****P < 0.0001, **P < 0.005, *P < 0.05 statistical analysis using two way annova. The dot plot clearly shows the cells are Annexin V FITC and PI positive cells under SM for all cell lines tested (**c**,**f**,**i**) compared to control cells (**b**,**e**,**h**).

**Figure 4:**
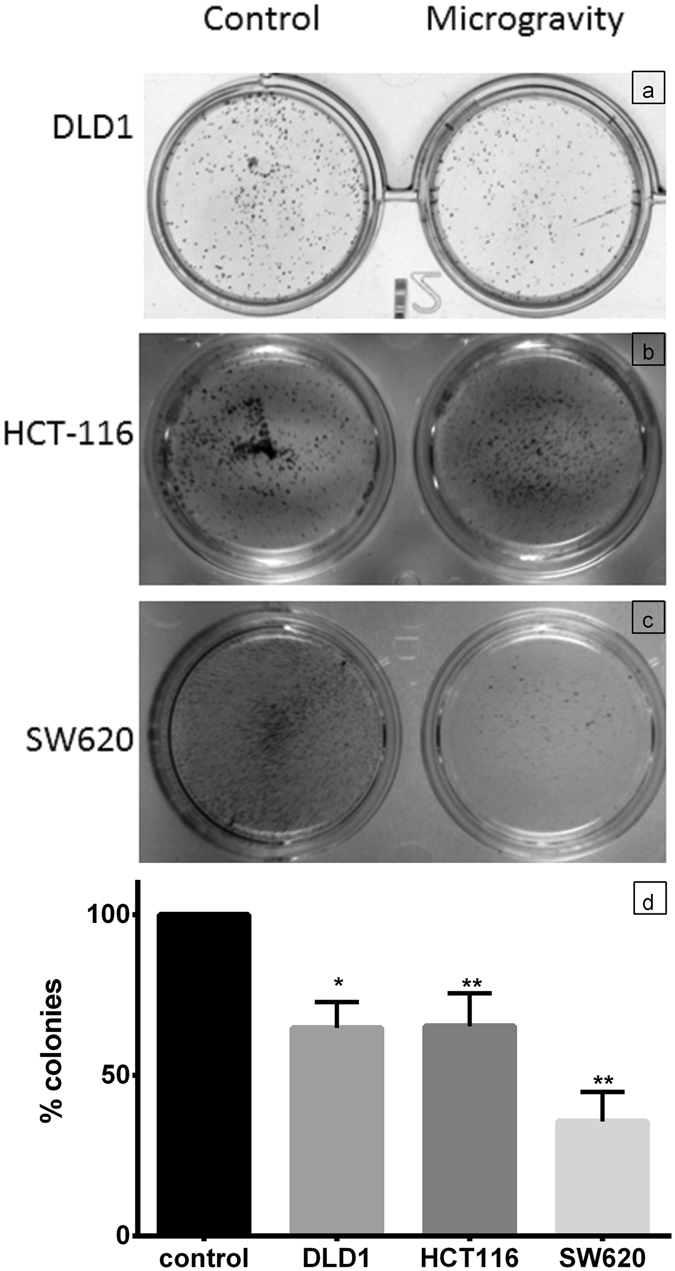
Cell growth is hindered with SM which results in reduced colony formation. The phase contrast image of colonies formed with 1000 cells in a 24 well plate for control and SM cells of DLD1 (**a**), HCT116 (**b**) and SW620 (**c**) show the reduced number of colonies in simulated microgravity. The data representation as mean + S.D. (**d**) depicts the reduction in percentage of colonies formed. The experiment was repeated thrice, data shows that colony formation was significantly reduced. P < 0.005 for HCT 116 and SW620, while P < 0.05 for DLD1.

To identify the mechanism of cell death we analyzed the Annexin V FITC, propidium iodide (PI) stained CRC cells under SM through Flow Cytometry, compared with control. There was significant early and late apoptotic population in cells under SM. Necrotic population of ~10% in DLD1 and HCT116 cells while ~20% in SW620 cells were also observed (Fig. 3). This may be due to hypoxic core existing in the large clumps and spheroids. The reduction in cell growth extended when the SM cells were shifted to normal gravity. These cells had lower colony forming capability (Fig. 4a–d) with SW620 cells greatly affected as compared to DLD1 and HCT116 cells. The DLD1 and HCT116 cells recovered growth rate when transferred to normal conditions, providing the right platform to study the molecular effects of the microgravity.

In our previous study, mRNA levels of cell cycle genes *CDK1, CDK2, CCNB1 and CCNE1* diminished in DLD1 subjected to SM^12^. To further understand the underlying molecular mechanism, we focused on DLD1, and studied the expression of specific cyclins and cell cycle inhibitors in the control of cell cycle in microgravity, we performed qPCR on DLD1 cells subjected to SM and shifted to static condition for 4 days (SS) revealed the Cell cycle inhibitors *CDKN2B* (p1INK4b) and *CDKN2D* (p16INK4d) were significantly higher in the SM. *CDKN2D* expression was maintained, whereas *CDKN2B* was downregulated when clumps were shifted to normal condition (Static Shift-SS). *CDK7* involved in the progression of cell cycle is downregulated in microgravity and maintained through the shifted condition. Cyclins *CCNG2* and *CCNT2* were upregulated in microgravity and in shifted condition (Fig. 2h).

These data confirm that cell death of colorectal cancer cells in simulated microgravity, mostly through activation of cell cycle inhibition pathways, leading to apoptosis. Moreover, gene expression profiling in DLD1 correlates with the growth reduction and apoptosis seen in SM.

### Adaptive responses of DLD1 to microgravity

**Figure 5:**
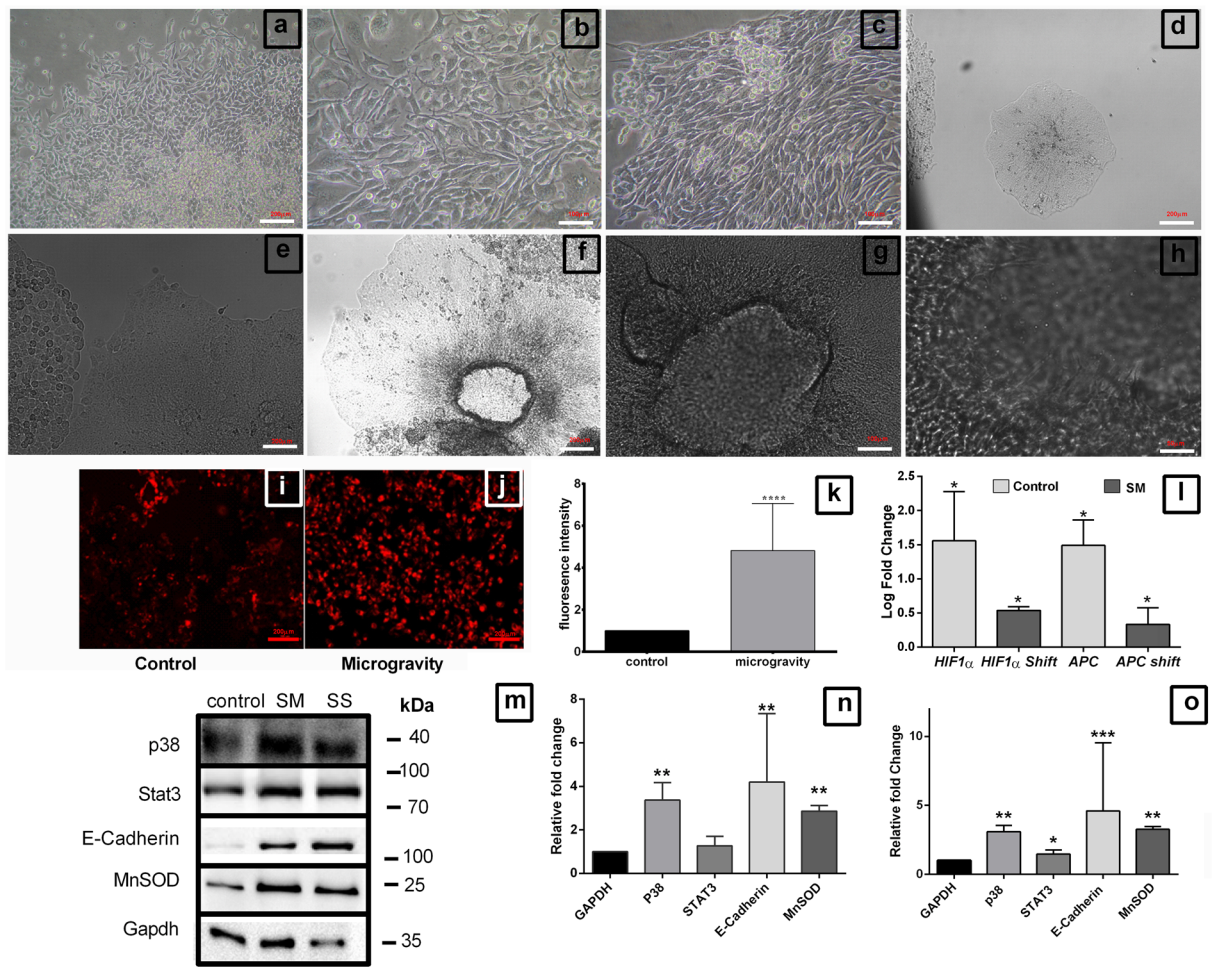
Adaptive responses of DLD1 to SM through differentiation. The morphogenetic effects of SM induced cell clumping, when clumps were shifted to normal culture conditions ((**a**–**c**) 2 weeks, (**d**,**e**) 4 weeks, (**f**–**h**) 2 months). The DLD1 cell clumps from SM, when shifted to normal conditions adhered to plate and formed colonies (scale = 200 μm) (**a**) which in two weeks, housed large and multinucleated cells, also cell-cell communication was profound with cell linkages (scale = 100 μm) (**b**) also some of the cells were polarized directionally (scale = 100 μm) (**c**). On prolonged culture for up to 2 months the specific features from the niche were more evident, some cells seems to retain the 3D conditioning and form rare, tightly packed colonies by 4 weeks (**d**) (scale = 200 μm), (**e**) (scale = 100 μm) which allows the cells to grow in a 3D fashion, forming cylindrical structures (**f**) (scale = 200 μm), these cells continuously aggregate and form large spheroid like formations (**g**) (scale = 100 μm) with formation of complex structures, facilitated through appendages (**h**) (scale = 50 μm). Mitotracker-red imaging showing the increased membrane potential in microgravity (scale = 200 μm) (**i**,**j**) and its graphical representation (**k**) P < 0.0005. Gene expression analysis of hypoxia related *HIF1α* and *APC* (Adenamatosis Polyposis Coli) in SM and static Shift (SS) compared against *GAPDH* as control (**l**). Data represented as mean + S.D. Western blot for proteins involved in adaptive response STAT3, P38 MAPK, MnSOD and endothelial marker E-Cadherin in SM and Shift condition (**m**) and the representative graph compared relative to GAPDH for SM (**n**) and SS (**o**). Experiment was performed thrice and data represented as mean + S.D. *P < 0.05, **P < 0.005, ***P < 0.001, statistical analysis done using Mann Whitney t test.

3D environment allows cells to exhibit phenotypic and genotypic characteristics of *in-vivo* condition minimally. The growth rate is reduced in 3D environment compared to 2D tissue culture plate owing to the increased physical constraints for cellular expansion. The reduced cell growth under SM was rescued when the DLD1 clumps were shifted to normal gravity and started to form colonies within two weeks’ time (Fig. 5a). Moreover, these cells exhibited extensive cell-cell communication through cell-cell bridges (Fig. 5b). A significant number of large multinucleated cells were observed in these cultures which were absent in the control (Fig. 5b). Some cells among the shifted clumps were polarized and directional, and tightly packed (Fig. 5c). These structural features were absent when the clumps were trypsinized and seeded onto tissue culture plate and cultured for the same period of time (data not shown). On prolonged culture over a period of two months with subsequent passaging, we observed that some rare cells among SS cells formed tight colonies (Fig. 5d), unlike other microgravity simulated cells, which eventually formed elaborate 3D structures (Fig. 5d–h). Small appendages seem to aid in the formation of this cellular alignment.

Adherent tumor cells live in an ischemic environment which has a hypoxic core and mitochondrial hyperpolarization. We examined the hypoxia inducible factor 1 alpha expression level using qPCR and mitochondrial membrane potential using Mitotracker-red fluorescence imaging to evaluate the presence and role of hypoxia in the observed morphogenic changes of the cells. As suspected the 3D clumps formed in SM housed a hypoxic core evident from the elevated *HIF-1α* expression and hyperpolarization of the mitochondrial membrane evident from Mitotracker-red staining (Fig. 5i–l). Adenomatosis polyposis coli (*APC*), involved in the 3D arrangement of cells is upregulated in microgravity and shifted condition ascertaining the very nature of these cells to sustain a 3D niche.

**Figure 6:**
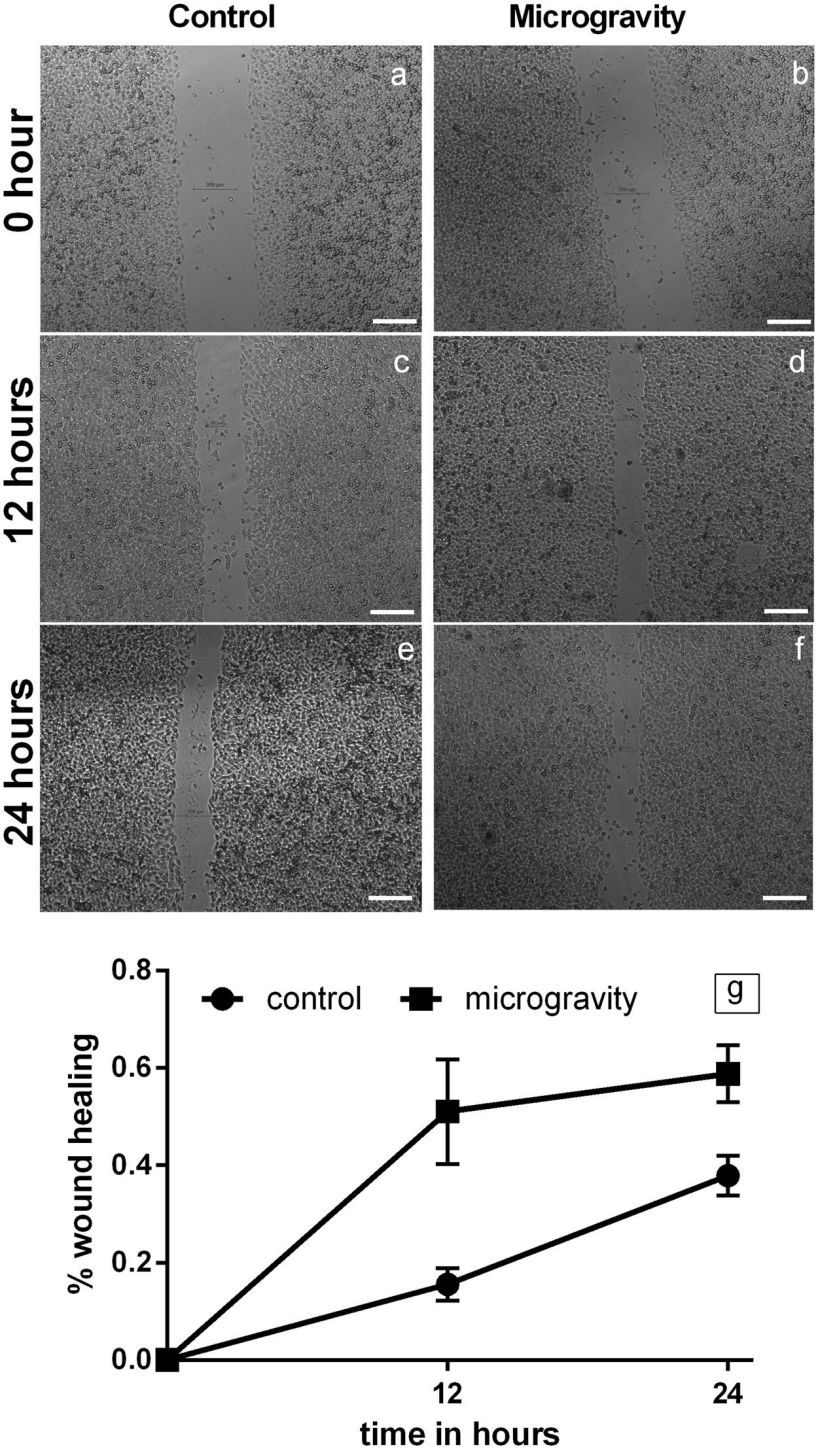
Microgravity increases cell migration. Phase contrast image of DLD1 control cells immediately after scratch from a T200 tip (**a**), 12 h post scratch wound (**c**) and 24 h post scratch wound (**e**). Comparative phase contrast image of SM DLD1 2 weeks post shifting to normal gravitational condition, 0 h (**b**), 12 h (**d**) and 24 **h** (**f**), scale = 200 μm. The representative graph (**g**) depicts 57.7% wound healing in SM after 12 hours, compared to 15.5% in control. The wound healing was 59% after 24 hours in SM compared to 38% in control.

For further analysis of adaptive responses, we performed western blots for stress response elements such as p38 MAPK, STAT3, cell-cell contact protein E-Cadherin and MnSOD. The expression levels were high compared to control and maintained through SS (Fig. 5m–o). We performed wound healing scratch assay on prolonged shift cells to ascertain the functional extension of the stress response in the DLD1 cells subjected to microgravity. The SM subjected cells clearly had a migratory phenotype with significantly faster wound healing compared to control (Fig. 6). These data strongly support that adaptive measures are triggered under microgravity, which was evident when the cells were shifted to normal conditions.

### FOXO3/PTEN/AKT axis determines outcome of cells subjected to SM

**Figure 7:**
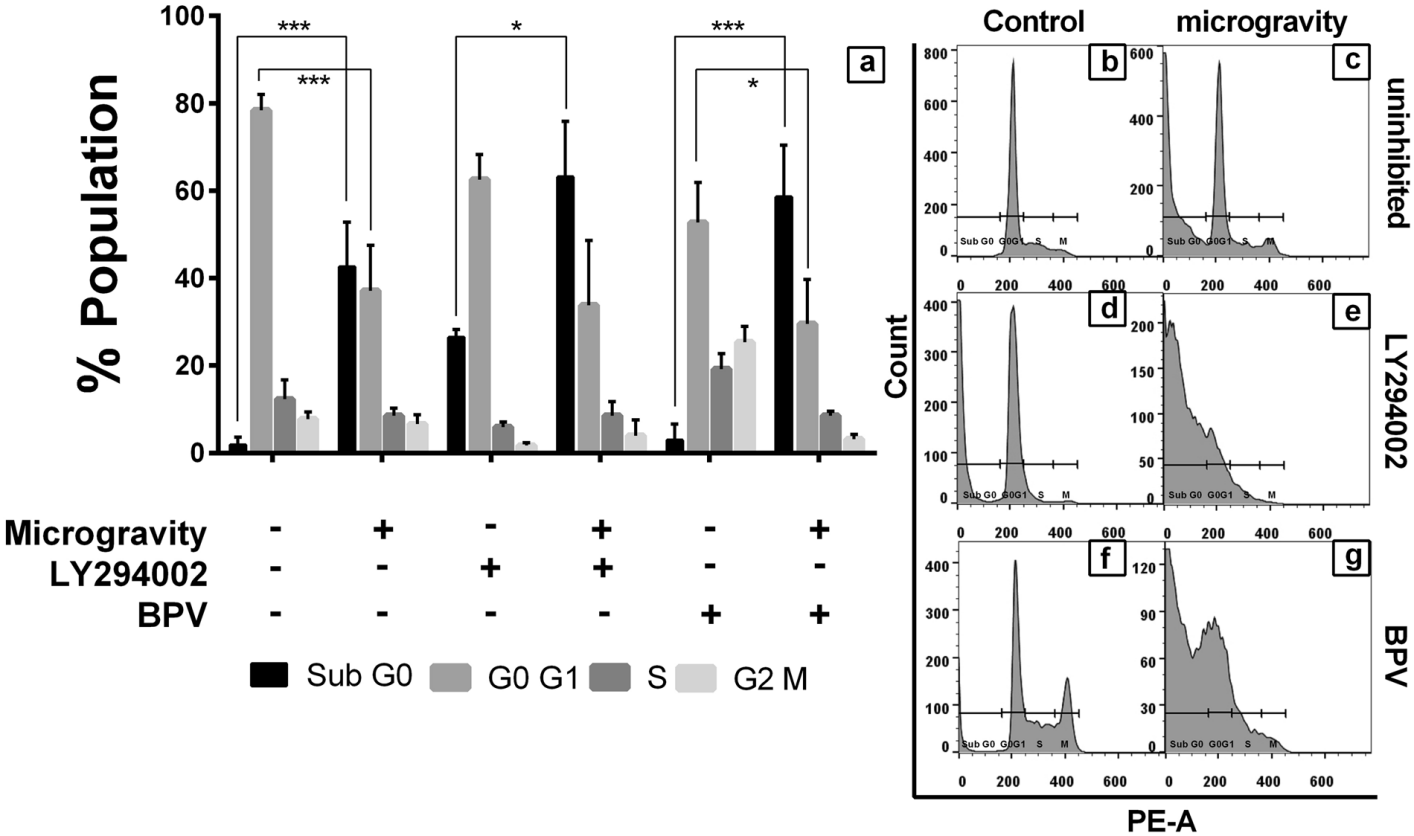
Cell cycle analysis of AKT/PTEN inhibited cells subjected to SM. The graphical representation of the population distribution of DLD1 cells across various stages of cell cycle during SM and SM after inhibition of AKT (LY294002 10 μM) or PTEN (bpV(HOpic) 14 nM) (**a**). The data is represented as mean + S.D. The representative histogram of PI intensity for control DLD1 (**b**), SM (**c**), AKT inhibition (**d**) and SM after AKT inhibition (**e**), PTEN inhibition (**f**) and SM after PTEN inhibition (**g**) shows clear shift in the PI intensity with each treatment.

Microarray analysis of DLD1 under SM had significant modification in AKT related pathways, suggesting possible canonical/non-canonical intervention of the pathway. PTEN a major inhibitor of AKT activation through inhibition of PIP2 to PIP3 transition was also upregulated in the microarray^12^. To elucidate the mechanism involved, we inhibited AKT activation through PI3K inhibitor LY294002 in DLD1 and analyzed cell cycle during control and SM conditions. Simultaneously cells were also treated with PTEN inhibitor bpV(HOpic) and followed with SM and cell cycle analysis. AKT inhibition causes G2M arrest under normal and SM conditions, which was also seen in the bpV-SM group. Such cell cycle arrest is absent when cells were simulated with microgravity without any intervention (Fig. 7). These results suggest that PTEN might take part in the survival strategy of cells in microgravity.

**Figure 8:**
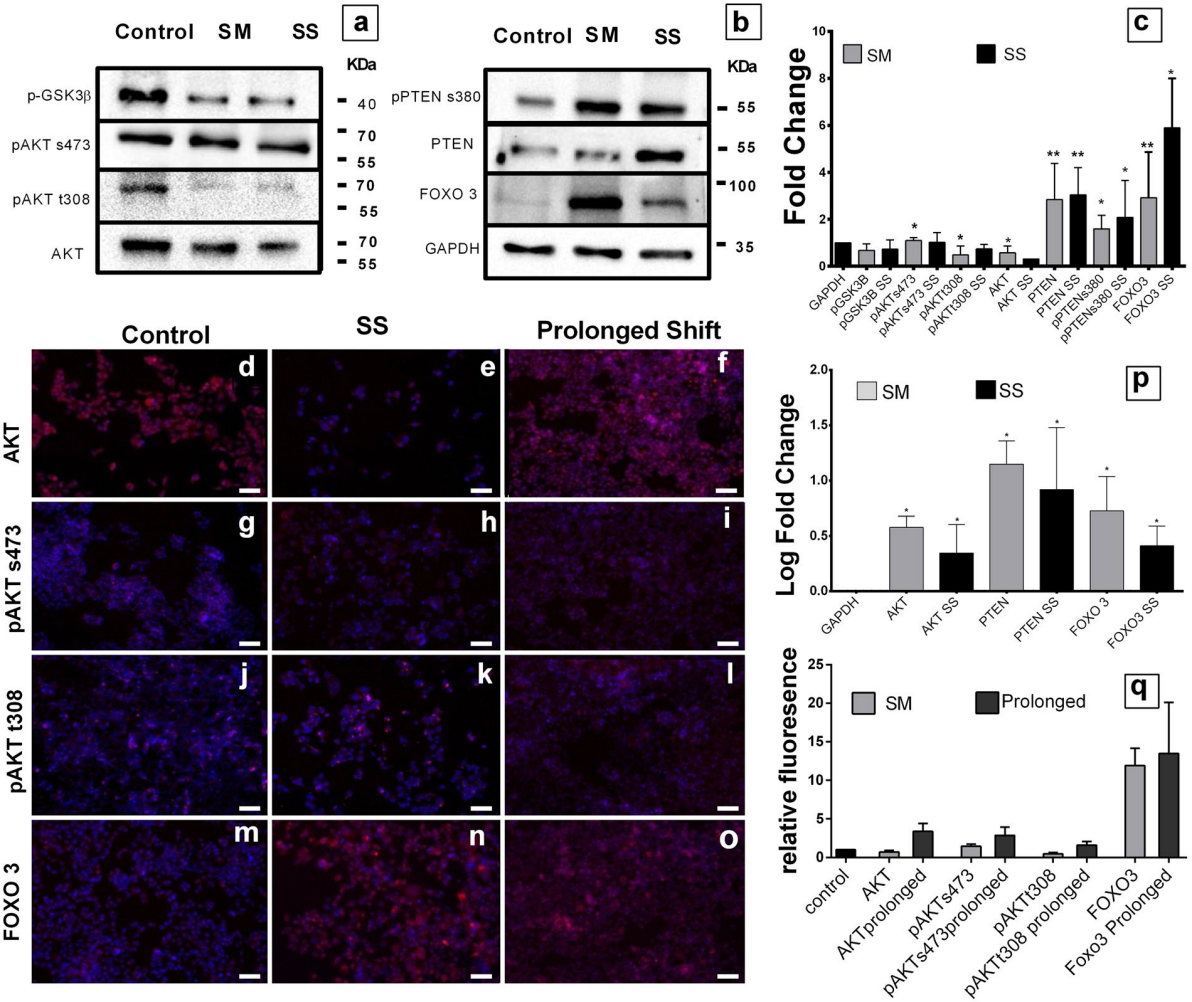
The PTEN/FOXO3/AKT axis regulation under microgravity. Images of Western blot for AKT pathway proteins; phosphorylated GSK-3β serine 9, phosphorylated AKTserine 473, phosphorylated AKT threonine 308 and total AKT of control, Microgravity simulated and SM cells shifted to normal gravity for 4 days (SS) (**a**). Western Blot images of phosphorylated PTEN serine 308, PTEN, FOXO3 and GAPDH (**b**). Representative graph normalized using GAPDH; black bars represent SS and lighter bars represent SM (**c**). Data represented as mean + S.D. statistical analysis using Mann Whitney t test *P < 0.05, **P < 0.005. Fluoresecnt images of DLD1 cells stained with PE conjugated secondary against AKT, pAKTs473, pAKTt308 and FOXO3, nucleus counterstained with DAPI, control (**d**,**g**,**j**,**m**) respectively and SS cells (**e**,**h**,**k**,**n**) and SM cells prolonged in static for longer period 2 months (**f**,**i**,**l**,**o**) scale bar = 200 μm. qPCR analysis of Gene expression of *AKT*, *PTEN, FOXO* genes against *GAPDH* as housekeeping in control vs SM and SS, represented in log fold change of mean + S.D. *P < 0.05 (**p**). Graphical representation for the images (**d**–**o**) showing relative fluorescence intensity between control, SS and prolonged shift cells (**q**).

We performed western blots and fluorescent imaging to assess the extent of the pathway modifications involved with microgravity. The protein level of AKT was diminished, along with the phosphorylated forms pAKTs473 and pAKTt308. In corroboration, the phosphorylated form of GSK-3β, a marker for the progression of AKT pathway, levels were also diminished. PTEN, its phosphorylation at serine 380 and FOXO3 were upregulated during microgravity and maintained under shift condition (Fig. 8a–c). Interestingly the mRNA levels were not giving the same picture. *AKT, PTEN* and *FOXO3* were upregulated and maintained through SM and SS (Fig. 8p). This supports a conclusion that cellular adaptive responses under SM are mediated by regulations in the protein turnover, rather than mRNA expression. Fluorescence imaging of SS DLD1 showed a similar scenario as western blots. AKT and its phosphorylated forms were downregulated in microgravity whereas FOXO3 was significantly high. But the prolonged culture of SS showed upregulation of AKT, phosphorylated forms and FOXO3 significant than the control cells housing a different scenario than the SM cells (Fig. 8d–o). Thus, indicating the changes was specific to SM. With our findings, it is clear that the SM alters the growth/differentiation control elements FOXO3/PTEN/AKT. The downregulation of AKT, a cross-play between FOXO3 and PTEN, results in the observed cell death and differentiation.

### Autophagy flux is altered in simulated microgravity

To elucidate the functional scenario of the molecular expression we studied the process of autophagy through LC3B and p62 western blots. LC3B II levels were critically increased in SM indicating autophagosome formation. But p62 (sequestrome1) was high in SM indicating problems in the maturation of the formed autophagosomes (Sup. Fig. 2a). Acridine orange stained SM and control cells under FACS analysis showed that both acidic vesicles (Phycoerythrin (PE)) and autophagic vesicles (FITC) were high in SM compared to control (Sup. Fig. 2b,c). The mRNA levels of autophagy controlling genes; analyzed using qPCR was also showcasing the same concept. The genes involved in autophagosome formation (*ATG4B, ATG7, ATG16L1, Beclin1, and LC3B*) were high in SM; conversely the genes involved in autophagosome maturation (*ATG5* and *ATG12*) were downregulated (Sup. Fig. 2d). These findings suggest that the FOXO3 dependent destructive mechanism of the cell, autophagy is activated but not matured in SM.

## Discussion

The immediate environment of the cell affects majority of cellular events and cell fate. Microgravity adversely affects various biological events, with various specific functional modifications observed in ground experiments and in space^17, 18^.

The cell death and growth arrest of cancer cells in simulated and actual microgravity are well documented in various cell types^5–7^. Our results were in corroboration with previous reports of apoptosis mediated cell death in CRC cells studied. The reduction in cell cycle during microgravity is mainly attributed to the disruption of the cytoskeletal elements resulting in G1 arrest^19^.

Our results clearly show a shift of the pathways involved in cell growth towards quiescence and differentiation when CRC cells were subjected to SM. Molecular analysis revealed downregulation of AKT mediated growth mechanism and upregulation of PTEN/FOXO3 mediated stress survival, in DLD1 cells under SM. The PTEN/FOXO3/AKT axis is a main regulator of the fate in cancer cells. The complete removal of functional PTEN is seen in advanced stage of cancer, especially gall bladder, prostrate and colorectal cancer^20–22^.

But recent evidences show that the role of PTEN and FOXO extend beyond the well understood canonical tumor suppressive ones. PTEN regulates reactive oxygen species (ROS) mediated cell senescence and DNA damage, and in its absence, the supporting effect of premature senescence to apoptosis is averted^23^, also the nuclear localization of PTEN is governed by redox stress^24, 25^. FOXO is pinned for its regulation of cancer stemness^26, 27^. The FOXO family proteins take over metabolism control in the absence of functional AKT, and determine cell fate^28^.

Involvement of FOXO in microgravity is imminent, but less explored and understood. Muscle atrophy is normally associated with space travel. This condition is critically dependent on FOXO activation and its subsequent effects on autophagy^29^. Hyun W. Ryu *et al*.^30^ observed increased autophagy under SM. Upregulation of AKT and subsequent migration and tube formation were seen in HUVECS subjected to clinorotation^31^. In random positioning of MDAMB-231, AKT is downregulated in clumps and upregulated in cell that did not form clump as compared to control^32^. These contrasting results make the role of AKT in microgravity unclear. AKT is known to be upregulated under hypoxia. The formation of spheroid induces hypoxia^33^, but inverse is observed in microgravity proposing a cytosolic regulation over the AKT mediated growth pathway.

We observed that the overall pathway of AKT is altered. Total and phosphorylated forms of AKT as well as the phosphorylated form of GSK-3β were downregulated. AKT promotes cell proliferation through phosphorylation of GSK-3β at serine 21 and its subsequent inhibition^34^. The downregulation of AKT pathway is accompanied with increased PTEN expression (canonical inhibitor of AKT activation) and its activated form pPTENs380. Subsequently FOXO3, (nuclear antagonist of AKT) is also overexpressed. Interestingly, the expression at the protein level is not supported at transcriptional level. There was constant overexpression of *AKT* mRNA along with *PTEN* and *FOXO3*, proposing an environment for *AKT* expression and function. Inhibitor studies enlightened that the observed G0 population was merely an additive effect of AKT inhibition present in both normal gravity and SM, compared to uninhibited cells under SM. Whereas, PTEN inhibition showed a contrasting effect between normal and SM in Sub G0 population. Significant G2-M phase arrest under AKT or PTEN inhibition followed by SM and not under normal gravity was seen. This G2M arrest was not present in uninhibited SM cells. This shows PTEN mediated regulation over AKT takes the center stage under microgravity survival. These observations lead to hypothesize that, the physiological effects of microgravity are mediated through FOXO/PTEN/AKT axis and is proven from our study.

We have earlier observed an overall reduction in cell cycle genes in DLD1 subjected to SM^12^. In the current study, we showed that CDK inhibitors CDKN 2B and 2D were highly upregulated in SM. The *CDKN2B* is a tumor suppressor gene, working in absence of p16INK4a^35^. It is also a regulator of fibrillar collagen induced tumor cell cycle arrest^36^. Its high expression in SM is in accordance to higher stress fiber under SM. FOXO 3 regulates the activation and of p15INK4b and p19INK4d, also AKT inhibition through PI3K inhibition causes p15INK4b & p19INK4d expression instead of p16INK4a and p18INK4c expression^37^.

*CDK7*, a control element between proliferation and differentiation, is required for the progression of cell cycle. It is expressed ubiquitously among multiple cancer types during all cell cycle phases, even in quiescent cells^38^. *CDK7* inhibition is required for adipocyte differentiation^39^. The down regulation of *CDK7* under SM and Shift can be directly reasoned for the observed cell death and morphogenetic changes.

*CCNG2* is exclusively expressed in quiescent cancer cells; *CCNG2* transfection reduces survival of prostate cancer cells^40^. Metformin induced AMPK activation and reduction of cancer risk requires *CCNG2* ^41^. *CCNG2* upregulation requires *FOXO3a* ^42^. The overexpression of *CCNG2* and *CCNT2* correlates with the reduced cell growth and colony formation capability. *CCNT2*, a regulatory subunit of p-TEFβ (a transcription mediator)^43^, was upregulated in microgravity and shifted cells. These can be the basis for reduced cell cycle under SM and observed features of differentiation in the shifted cells.

The simulation of microgravity induces characteristic features as seen in *invivo* conditions. Ovarian and prostrate tumor 3D models and *invivo* mimics are attained using rotating wall vessel (RWV) cultures^44, 45^. The modified physical forces of SM create a niche for cells to interact and form spheroids/clumps which when shifted to normal conditions exhibit features of morphogenetic changes and 3D structures. These structure formations are an extension of 3D conditioning under SM and are unique to 3D cultures, such features are lost when spheroids are trypsinized and seeded as single cell suspension. Similar observations have been made on MCF7 cells under SM using RPM where 3D duct-like glandular structures were formed^46^. E-Cadherin expression was induced in SM and might be associated with increased PTEN expression and 3D morphogenetic changes^47^.

Apart from microgravity, hypoxia and shear stress have a significant impact under SM. The observed polarization of cells may be a direct result of the recurrence of normal shear when shifted from low shear conditions of SM. Shear stress induces PTEN-AKT dependent cellular polarization in vascular endothelial cells^48^. Doolin E. J. *et al*.^49^ found that microgravity is associated with increase in cell proliferation and nuclear size without changes in cytokeratin levels. Further the study suggested a role of loss in shear forces and increase in nutrient bioavailability in influencing these changes.

Hypoxia facilitates differentiation^50^ and is associated with cancer stemness^51, 52^. The elevated levels of *APC* and *Hif1-α* in our SM experiments are in accordance with the formed 3D environment and hypoxic core respectively. APC interacts with E-cadherin and an important regulator of WNT signaling, playing a major role in CRC stemness^53^ we observed *WNT* genes upregulated in microarray in our previous study^12^. Moreover, upregulation of MnSOD and P38 MAPK (which are involved in cellular ROS response) shows that cellular adaptive mechanisms via MAPK are also active. STAT3 activity is anti-tumorigenic through senescence in the presence of PTEN^54^ and is pro-tumorigenic under HDAC inhibition^55^ showing a cross-play between these proteins on stress survival. Recent evidences show that stress response in cancer results in adverse effects like resistance development and activation of differentiation^56^.

Microgravity has been shown to facilitate migration. Rat BMSCs subjected to clinorotation has shown increased functional recovery from spinal cord injury, through migration of the simulated cells^57^ Eahy926 cells under simulated microgravity had 25% more migration compared to cells grown in static 1 g, this migration was inhibited by cytochalsin D^58^. The over-expressed proteome of E-Cadherin, p38 MAPK and FOXO3 promotes migration^59, 60^. The adaptive responses to the physical stress and the expressed proteome reflect in the form of wound healing. SM DLD1 cells exhibited significant increase in wound healing than control. Stiffness of the substratum is also a major factor for migration^61^ SS condition also involves a change in the stiffness of the substratum aiding the process.

There is a positive relation between the clump formation and cell survival. DLD1 and HCT116 cells were better adaptive than SW620 forming bigger spheroids as reflected in number of colonies formed in colony forming unit (CFU) assay. Our observed morphological and functional features suggest a close relation to *in vivo* tumor and a possibility for mimicking the tumor niche *in vitro*.

The autophagy flux requires fusion between autophagic vesicles and lysosome. However, the elevated levels of distinct FITC and PE stained vesicles in SM indicated that the fusion is hampered. This also correlates with the increased LC3BII formation and p62 accumulation. The fusion of autophagosome and lysosome requires its interactions with microtubules through dynein and kinesins^62^. The activation of FOXO3 may facilitate the formation of autophagic vesicles, but the disruption of the cytoskeleton, promptly observed in microgravity^19^, makes autophagy flux less feasible.

Collectively with this information we understand that, the DLD1 cells react to change in gravitational stress with reduced cell growth and increase in autophagy. But, the removal of the stress results in adverse implications like 3D morphogenetic alignment, increased wound healing, and presence of proteome that supports proliferation and differentiation alike.

## Conclusion

Our results highlight the role of PTEN/FOXO3/AKT axis in the control of cell fate during and after SM. The cell death under SM is mediated through CDKNs upregulation through tumor suppressors FOXO3 and PTEN. This is the first evidence of upregulation of PTEN under the influence of change in gravitational forces. Here we found a deregulation in the autophagy flux under simulated microgravity, further analysis can lead to better understanding of the role of physical factors that control autophagy flux. The effect of stress removal on the DLD1 cells resulted in complications of adaptive measures including migration and morphogenetic differentiation, two key features of metastatic cancers. Understanding this stress response mechanism in cancer, especially the role of tumor suppressors under stress and stress removal is essential for traversing hurdles of resistance development. Confirmation of the same pathway in disease free model can further lead to intervention of deleterious effects of space travel.

## Materials and Methods

### Cell Culture

Colorectal cancer cell lines DLD1 (Duke’s type C colorectal Adenocarcinoma), HCT 116 (human Colorectal carcinoma), and SW620 (Human Caucassian Adenocarcinoma) were procured from National Centre for Cell Science, Pune, India. DLD1 cells are isolated from primary tumor loci whereas SW620 is derived from lymph node after metastasis. HCT116 is a metastatic cell line. The cell lines were maintained in RPMI 1640 medium (Thermo Scientific, USA) supplemented with 10% Fetal Bovine Serum (Thermo Scientific, USA) and 10 U/ml & 10 μg/ml Penicillin and Streptomycin (Thermo Scientific, USA) respectively. The cells were subjected to microgravity under logarithmic growth conditions. For Pi3K and PTEN inhibition, the cells were treated with LY294002 (Sigma Aldrich, USA) and bpV (HOpic) (Sigma Aldrich, USA) at IC_50_ for its activity at 10 μM and 14 nM respectively for overnight in serum free medium before subjecting to microgravity.

### Simulation of Microgravity

The colorectal carcinoma cells were trypsinized and seeded into High Aspect Ratio Vessel (HARV) with a Rotating Cell Culture System RCCS (Synthecon, USA) at a seeding ratio of 2 × 10^6^ cells/10 ml in growth media. The HARV was operated at 10 RPM for 48 hours, the induction of microgravity was observed with lingering clumps of cells which indicated simulation of microgravity (SM). The media was replaced every 16–24 hours, and additional media was added periodically to avoid air bubble formation or foaming. The clumps formed were harvested without excessive force using a large mouth Pasteur pipette. The clumps were either transferred directly for morphogenetic studies or dissociated using 0.25% Trypsin-EDTA (Thermo Scientific, USA) to form monolayer in tissue culture plates and cultured in normal gravitational conditions termed as static shift (SS). The shifted cells were cultured for 4 days prior to cell cycle, apoptosis and gene expression analysis and 7 days for colony forming unit analysis. The Shifted clumps were maintained for two months for observations and expression analysis (prolonged shift). The shifting of SM cells to normal conditions provides a platform to study the effects of stress removal, especially in terms of gene expression.

### Viability Assay using XTT

To analyze the cell death induced by microgravity, 5 × 10^5^ CRC cells were seeded into a 2 ml HARV and subjected to microgravity in RCCS culture. The whole volume was centrifuged at 1000 RPM and dissociated in 0.5 ml culture media, and incubated with XTT (2,3-Bis-(2-Methoxy-4-Nitro-5-Sulfophenyl)-2H-Tetrazolium-5-Carbox-anilide) (Himedia, India) solution at 0.25 mg/ml final concentration for 3 hours at 37 °C. For control, CRC cells were trypsinized and 5 × 10^5^ cells were counted and treated with XTT. The color formation was quantified at 490 nm wavelength in a microplate reader after transferring to a 96 well plate.

### Cell Cycle Analysis

The cells were harvested by trypsinization and counted. 1 × 10^5^ cells were washed and resuspended in DPBS and fixed in ice cold ethanol under constant agitation to avoid clumping, to a final concentration of 70%. The cells were fixed for 30 minutes in 4 °C, washed in DPBS and centrifuged at 1000 RPM for 5 minutes. The fixed cells were incubated with RNase and PI (10 μg/ml and 50 μg/ml respectively) for 30 minutes at 37 °C followed with population distribution of cell cycle analysis in BD FACS Canto flow cytometer (BD, USA).

### Apoptosis Assay

1 × 10^5^ cells from control, microgravity simulated (SM) and cells shifted to static conditions for 4 days (SS) were counted and washed in DPBS. The cells were pelleted and re suspended in binding buffer and followed with incubation with AnnexinV conjugated FITC and PI as per the manufacturer’s protocol (BD APOPTOSIS assay kit). The population distribution of live and dead cells was analyzed in BD FACS Canto flow cytometer.

### CFU Assay

Single cells suspension of control cells and microgravity subjected cells were seeded at a rate of 1000 cells per well in a 24 well plate, and grown for 7 days in RPMI 1640 media supplemented with 10% Fetal bovine serum and 2% horse serum, in 37 °C, 5% CO_2_, >95% humidity incubator. The colonies formed are counted after staining with crystal violet (0.5% in 25% methanol w/v) for 30 minutes, air dried and washed with DPBS. The colonies were visualized in Nikon eclipse Ti phase contrast microscope. Any group with more than 50 cells is accounted as a colony and counted.

### RNA Isolation and cDNA Conversion

The cells were collected after subjecting to microgravity, through centrifugation and washed in DPBS. The total RNA was isolated from 1 × 10^6^ cells lysed in TriZol (Sigma Aldrich, USA), and the total RNA was isolated from the aqueous fraction, following the manufacturer’s protocol. 2 μg of total RNA was converted into cDNA using MMLV-RT (Thermo Scientific, USA) and Oligo-dT (NEB, USA). The converted cDNA is used for gene expression analysis.

### qPCR

The gene expression pattern between the control cells, cells subjected to microgravity and those that were transferred to normal gravitational conditions was performed using SYBR (Thermo scientific, USA), in an 7500 Real Time PCR System (Applied Biosystems, USA). The fold change of gene expression was calculated from the ΔΔCt using pfaffl spread (http://www.microbiologybook.org/pcr/pcr-pfaffl.htm) using *GAPDH* as house-keeping gene. The primer list is provided in (Supplementary Table 1).

### Protein Isolation

Protein was isolated from the cells in (Radio ImmunoPreceipitationAssay) RIPA buffer containing 10 mM Tris –Cl, 1 mM EDTA, 0.5 mM EGTA, 1% TritonX-100, 0.1% Sodium deoxycholate, 0.1% SDS, 140 mM NaCl, pH 8.0 supplemented with 1X Protease Inhibitor Cocktail and Phosphatase Inhibitor Cocktail (Sigma Aldrich, USA). The cells were scrapped in ice cold DPBS, centrifuged and then incubated in lysis buffer at 4 °C with constant agitation in a rotospin (Tarsons, India) at 20 RPM. The isolated protein is centrifuged at 10,000 × g to remove debris, and the supernatant was quantified using BCA protein estimation kit (Thermo Scientific, USA). The protein was solubilized in sample solubilizing buffer (SSB) for immunoblotting or used as such for immunoprecipitation. The lysate was snap-freezed in liquid nitrogen and stored in −20 °C refrigerator for storage.

### Immunoblotting

Protein lysates were separated based on molecular weight by SDS PAGE (Sodium dodecyl sulfate Pol-acrylamide Gel Electrophoresis), and electro blotted to 0.22 μm Nitrocellulose membrane (NCM) (Pall, USA). The transferred NCM was blocked in Tris-buffered saline containing 3% Bovine Serum Albumin (Sigma Aldrich, USA) and 0.2% Tween-20 (Sigma Aldrich, USA) and incubated in primary antibodies for pan AKT, phosphor-AKTs473, phosphor-AKTt308, phospho-c-Raf, phospho-GSK-3β, PTEN, phospho-PTEN, phospho-PDK1 (Cell Signaling Technology, USA) for overnight at 4 °C and then washed with TBST buffer. The blots were then incubated in Horse-radish peroxidase conjugated secondary antibody raised against rabbit or mouse (Sigma Aldrich, USA) diluted at 1:10,000 in blocking buffer for 1 hour in room temperature. The blots were then washed with TBST followed with TBS and the protein presence was probed through enhanced chemiluminescence, using Westar Supernova (Cynagen, Italy) and documented in ChemiDoc + XRS (Biorad, USA). The developed bands were analyzed using Image lab 5.1(Biorad, USA).

### Mito-Tracker Red Staining

70% confluent plates of control and SM SS DLD1 cells were washed with DPBS to remove Serum and incubated with (50 nM) Mito-tracker Red CMX Ros (ThermoFischer, USA) for 20 minutes at 37 °C, 5% CO_2_ environment. The plates were washed with DPBS and imaged using Nikon eclipse Ti microscope (ex579 nm, em630 nm), and images processed as same as fluorescent images.

### Wound Healing Scratch Assay

The static shifted SM DLD1 cells (SS) were grown to 90% confluency in a 30 mm Petridish and compared against a 90% confluent control DLD1 dish. A wound is created using 200 μl tip and images were recorded in Nikon eclipse Ti phase contrast microscope (T0). At each time interval (12 h (T1) and 24 h (T2)) images were taken at different points across the wound. The difference in the cell free area between T0 and T1 or T2 is averaged across the different images using ImageJ software (https://imagej.nih.gov/ij/) to yield percentage wound healing.

### Fluorescent Microscopy

Control and SM DLD1 cells were seeded onto 24 well plate and grown for 60%–70% confluency. The cells were then washed in DPBS and fixed in 4% Paraformaldehyde, blocked, incubated with specific primary antibody at 4 °C overnight and Alexa Fluor 598 conjugated secondary antibody (Thermo Fischer, USA). The nucleus was counterstained using DAPI (300 nM). The fluorescence was recorded in Nikon eclipse Ti microscope (Nikon, Japan) at excitation (358 nm DAPI and 579 nm PE) and emission (460 DAPI and 630 PE) at (10X) resolution, using a Plan Fluor 10X DIC L objective and 0.3 numerical aperture. The images were captured in a camera. The experiment was performed twice and analysis was compared between 3 images between each well. All fluorescent Image analysis was performed using NIS-elements AR software package (Nikon, Japan).

### Autophagosome Staining using Acridine Orange

The SM cells were trypsinized and fixed in 4% paraformaldehyde and washed with DPBS, stained with Acridine orange (1 µg/ml) at room temperature for 20 min, washed with DPBS and the green and red fluorescence were analyzed in a BD. FACS Canto flow cytometer (BD. USA).

### Statistical Analysis

All Experiments were performed at least thrice, unless mentioned. The results are expressed in terms of mean + S.D. The Statistical analysis for the gene and protein expression was performed using Mann-Whiteney T test and Two-way annova for cell cycle analysis with 95% confidence interval (GraphPad Prism version 6.01, GraphPad software, La Jolla California USA).

## Electronic supplementary material

Supplimentary information
