# Supplementary material for: PTEN/FOXO3/AKT pathway regulates cell death and mediates morphogenetic differentiation of Colorectal Cancer Cells under Simulated Microgravity
Source: Sci Rep. 2017 Jul 20;7:5952. doi: 10.1038/s41598-017-06416-4 (PMC5519599; doi:10.1038/s41598-017-06416-4)
Supplement: Supplementary file 1 — Supplimentary information [file 41598_2017_6416_MOESM1_ESM.doc]

**PTEN/FOXO3/AKT pathway regulates cell death and mediates morphogenetic differentiation of Colorectal Cancer Cells under Simulated Microgravity**

Authors:

Raj Pranap Arun, Divya Sivanesan, Prasanna Vidyasekar^#^, Rama Shanker Verma*

Stem Cell and Molecular Biology Laboratory, Bhupat and Jyoti Mehta School of Biosciences, Department of Biotechnology, Indian Institute of Technology Madras.

# Present address- CSI-NUS Singapore

*Corresponding Author

Dr. Rama S. Verma

201, Department of Biotechnology

Bhupat and Jyoti Mehta School of Biosciences

Indian Institute of Technology Madras, Chennai, India, 600036

phone: 91-44-22574109,

e-mail: [vermars@iitm.ac.in](mailto:vermars@iitm.ac.in)

**Supplementary Figure1: Massive clumps of spheroids are formed after 48h of simulated microgravity in HCT116.**

Individual spheroids formed under SM aggregate and form massive clumps of ~0.5 cm.

**Supplementary Figure2: The progress of autophagy is disrupted in microgravity**.

Images of Western blot for Autophagy initiation and maturation proteins LC3B and p62 (a). The increase in autophagic vesicles seen from Acridine Orange staining of cells, red color represents microgravity and blue color control. The FITC histogram for autophagosome (b) and acidic vesicles (c).The log-fold change in mRNA level expression of genes involved in autophagy process, relative to *GAPDH* (d). The lighter bars represent SM and darker bars represent SS. Data representation as mean + S.D. the experiment was performed once and holds no statistical significance.

**Supplementary Figure 3:**

Uncut western blots used in the manuscript figures 5, 8 and Supplementary fig. 2

**Supplementary Table 1:**

List of Primers used

| **Gene Name** | **Primer Sequence** | |
| --- | --- | --- |
| *GAPDH* | Forward | GAC ACC ATG GGG AAG GTG AA |
|  | Reverse | GAT CTC GCT CCT GGA AGA TGG |
| *FOXO3* | Forward | CGC AGC CAA GAA GAA GGC A |
|  | Reverse | TGT AGA GCA TGG GCG AGA GA |
| *AKT* | Forward | GAA GCA GGA GGAGGAGGA GA |
|  | Reverse | TGG CCA CGA TGA CTT CCT TC |
| *PTEN* | Forward | GCT CAG TTC TCT CCT CTC GGA A |
|  | Reverse | AGG CTG CAC GGT TAG AAA AG |
| *CCNG2* | Forward | ACT TGG CAG GTC ATG AA |
|  | Reverse | CCG GGG TAG CCT CAA TCA AA |
| *CCNT2* | Forward | TGC ATC TCA CAA CCA CAC T |
|  | Reverse | CAG TTT CAC GAG GGT GCT GA |
| *CDK7* | Forward | TCG GGC AAA GCG TTA TG |
|  | Reverse | CCA AAA GCA TCA AGG AG |
| *CDKN2B* | Forward | CGG GGA CTA GTG GAG AAG GT |
|  | Reverse | GGG TGA GAG TGG CAG GGT |
| *CDKN2D* | Forward | CAT CTG GCA GTT CAA GAG GGT |
|  | Reverse | AGC CAC AAA CTG TGC TCC TC |
| *MAP1LC3B* | Forward | CGG TGA TAA TAG AAC GAT ACA AGG |
|  | Reverse | CTG AGA TTG GTG TGG AGA CG |
| *BECLIN* | Forward | GGA CAC TCA GCT CAA CGT CA |
|  | Reverse | AGC CTG GAC CTT CTC GAG AT |
| *ATG4B* | Forward | GCC GAG ATT GGA GGT G |
|  | Reverse | GCC TAT GGA CTT GCC TTC |
| *ATG7* | Forward | AGG AGA TTC AAC CAG AGA CC |
|  | Reverse | GCA CAA GCC CAA GAG AGG |
| *ATG16L1* | Forward | TCC AGG AGG CGG CAA G |
|  | Reverse | ATC AGA AGT TTC ATC CAC AAT G |
| *ATG12* | Forward | TCT ATG AGT GTT TTG GCA GTG |
|  | Reverse | ATC ACA TCT GTT AAG TCT CTT GC |
| *HIF1-α* | Forward | GCC AGATCT CGG CGA AGT AA |
|  | Reverse | CAG AAG TTT CCT CAC ACG CA |
| *APC* | Forward | TTT CCT TAC AAA CAG ATA TGA CCA G |
|  | Reverse | CGT GTA GTT GAA CCC TGA CCA |

**Supplementary Figure 1**


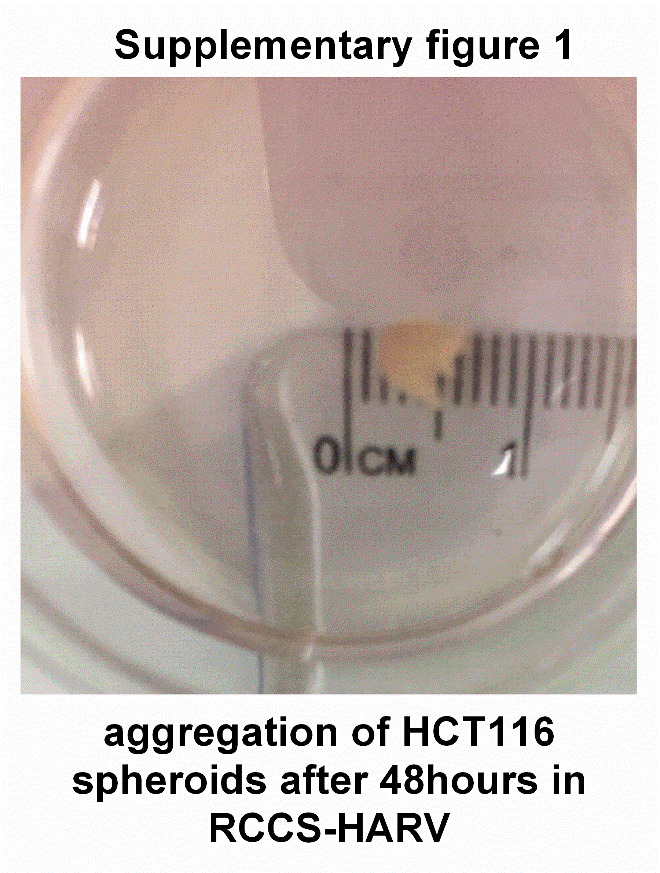


**Massive clumps of spheroids are formed after 48h of simulated microgravity in HCT116.**

**Supplementary Figure 2**


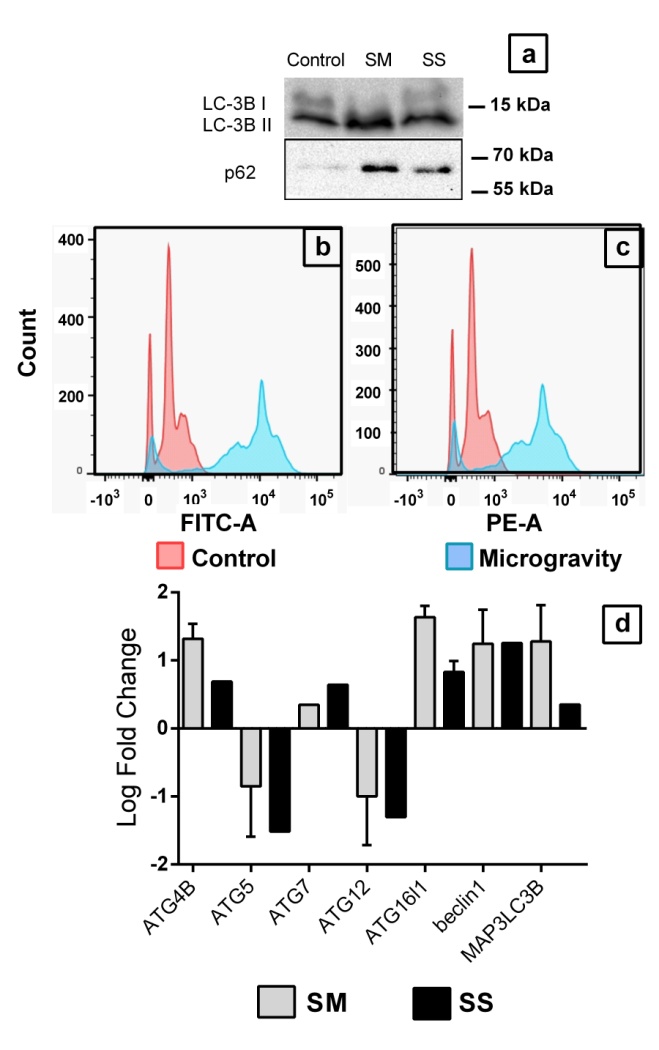


**The progress of autophagy is disrupted in microgravity**.

**Supplementary Figure 3**

**
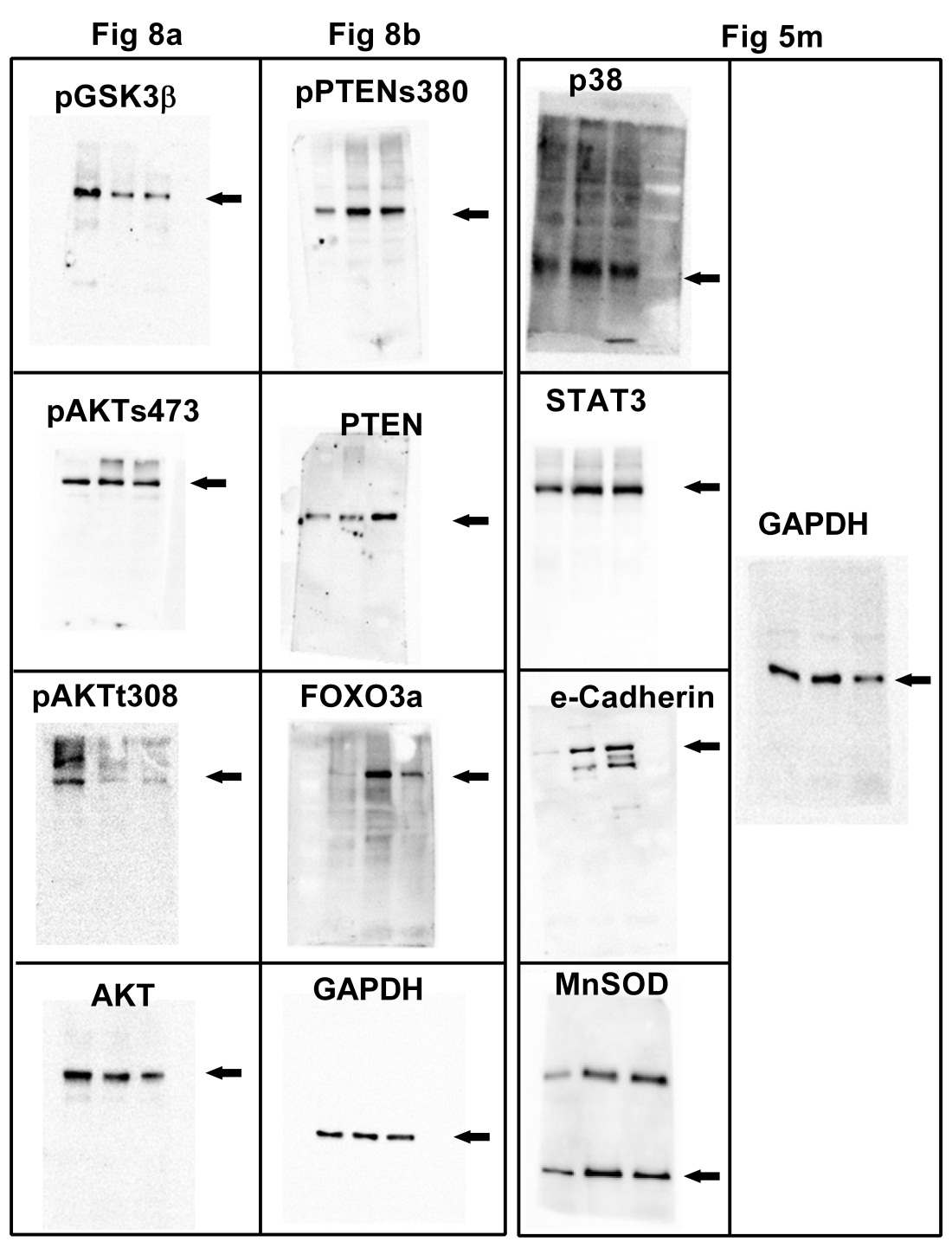
**

**Uncut blots used in the manuscript**
